# Exogenous Linoleic Acid Intervention Alters Hepatic Glucose Metabolism in an Avian Embryo Model

**DOI:** 10.3389/fphys.2022.844148

**Published:** 2022-02-21

**Authors:** Xiufen Zhang, Qilin Wu, Wenxuan Zheng, Chuang Liu, Liang Huang, Xin Zuo, Wenquan Xiao, Xiaofeng Han, Hui Ye, Wence Wang, Yongwen Zhu, Lin Yang

**Affiliations:** ^1^Guangdong Provincial Key Laboratory of Animal Nutrition and Regulation, College of Animal Science, South China Agricultural University, Guangzhou, China; ^2^Wen’s Food Group Co., Ltd., Yunfu, China

**Keywords:** avian embryo model, glucose homeostasis, linoleic acid, *in ovo* feeding, energy status

## Abstract

In the present study, developmental changes of gluconeogenesis and glycolysis in an avian model were measured, and then the intervention effects of *in ovo* feeding (IOF) linoleic acid (LA) on hepatic glucose metabolism were evaluated. In Experiment 1, thirty fertilized eggs were sampled on embryonic days (E) of 16, 19, 22, 25, 28, 31, and thirty newly-hatched ducklings at hatch (E34 and E35). In Experiment 2, a total of 120 fertilized eggs (60 eggs for each group) were injected into the yolk sac with PBS as the control group and LA as the IOF LA group on E25. Twelve eggs were selected for sample collection on E28 and E31. Serum contents of glucose, pyruvate, and lactate increased ( *p <* 0.05) linearly or quadratically from E16 to hatch, as well as hepatic glycogen and pyruvate contents. Hepatic mRNA expression related to energy homeostasis, gluconeogenesis, and glycolysis increased ( *p* < 0.05) in embryogenesis, and the plateau period was presented on E25–E31. IOF LA decreased ( *p* < 0.05) serum contents of glucose, triacylglycerol, cholesterol, and hepatic oleic acid, unsaturated fatty acids on E28, as well as the gene expression relative to gluconeogenesis. IOF LA increased ( *p* < 0.05) pyruvate content in serum and liver, and hepatic gene expression relative to glycolysis on E31. In summary, hepatic gluconeogenesis and glycolysis were enhanced to meet the increasing energy demands of embryonic development during E25 – hatch. Exogenous LA intervention on E25 could inhibit hepatic gluconeogenesis and enhance glycolysis during the later developmental period, disrupting glucose embryonic homeostasis and energy status.

## Introduction

The avian embryo derives all its nutrient requirements from the nutrient deposits in the fertile egg during incubation. Avian embryos make use of O_2_ accession for fatty acid (FA) oxidation to get energy with the vascular system and pulmonary development (Tazawa et al., 1983; Moran, 2007). However, energy metabolism shifts from yolk lipids to the predominant carbohydrate substrate when the embryo reaches the oxygen consumption plateau (Donaldson et al., 1991; De Oliveira et al., 2008). Thus, gluconeogenesis and glycolysis are enhanced during the late-hatch stage to maintain the energy homeostasis as well as normal physiological status for embryonic growth and development (Hu et al., 2017; Wan et al., 2018). For example, glycolysis reduces glucose to pyruvate and adenosine triphosphate (ATP), and pyruvate is converted to lactate that can be recycled back to glucose in the liver *via* the Cori cycle once the oxygen is available (De Oliveira et al., 2008; Bolaños et al., 2010). In addition, avian embryos are much easier to maintain and manipulate than most other vertebrate species. Therefore, it is speculated that an avian embryo during the last phase of incubation could be considered as an ideal model to understand vertebrate development *via* altering glucose homeostasis.

*In ovo* feeding (IOF) is an efficient technique to evaluate the effects of the exogenous nutrients on embryonic development using an avian model (Givisiez et al., 2020). IOF can deliver early nutrients and additives to embryos *via* yolk sac, albumen, air sac, and amnion et al. (Das et al., 2021). Previous studies have shown the effects of IOF carbohydrates, amino acids, minerals, and vitamins on embryonic development, and these exogenous nutrients can change blood histology, modify the regulation of transcription of different genes, and improve hatchability and perinatal growth in embryos and neonates (Tangara et al., 2010; Yair et al., 2015; Chen et al., 2021). In addition, IOF supplements like probiotics, prebiotics, and synbiotics protect against harmful gut microbes through competitive exclusion and maintain intestinal immune homeostasis (Shehata et al., 2021). There are limited reports on the effects of FA interventions. Linoleic acid (LA) has been proved to be involved in the regulation of glucose homeostasis *via* the alteration of FA and glucose metabolism *in vivo* and *in vitro* (Hamilton and Klett, 2021). Dietary LA could decrease blood glucose in obese rats (Matravadia et al., 2016; Holmäng et al., 2018). Glucose acts as an important fuel source for embryonic development (Minhas and Khan, 2016). Therefore, the developmental changes of serum glucose and hepatic glycogen concentrations in embryogenesis were measured to determine the critical period of glucose metabolism in the current study. We hypothesized that LA may decrease embryonic serum glucose and the adverse effects of IOF LA on the alteration of glucose metabolism to regulate embryonic development was evaluated using the avian embryo model.

## Materials and Methods

### Ethics Statement

The protocol was reviewed and approved by the Animal Care and Use Committee of South China Agricultural University with the following reference number: No. 20110107–1.

### Incubation and *in ovo* Feeding

Fertilized Muscovy duck eggs were obtained from a commercial breeder farm (Wen’s Food Group Co., Ltd., Yunfu, Guangdong, China), and all the eggs were collected from hens at 33 week of age belonging to the same breeder flock. The eggs were incubated in the automatic-controlled incubator (Dezhou Keyu Hatching Equipment Co., Ltd., Dezhou, China) according to standard hatchery practices (37.5 ± 0.5°C), relative humidity (55 ± 5%) until embryonic day 31 (E31). Next, all eggs were transferred to hatching crates and moved to hatchers which were set at a temperature of 37.0 ± 0.5°C and declined to 36.0 ± 0.5°C at the end of incubation.

In Experiment 1, a total of 350 Muscovy duck eggs were collected for incubation, and unfertilized and unviable eggs were discarded after candling on E15. A total of 180 viable eggs (75.2 ± 1.8 g) and 30 hatchlings (43.19 ± 0.7 g) were applied for the test (five embryos per replicate, *n* = 6). Thirty viable eggs were selected for samples collection on E16, E19, E22, E25, E28, and E31, and 30 ducklings at hatch (E34 and E35). In Experiment 2, a total of 200 eggs were collected for incubation, and 120 viable eggs (79.6 ± 1.9 g) were divided into two groups on E25, the control group and the treatment group, with 60 eggs each. The eggs in the control group or the treatment group were injected into the yolk sac with a volume of 100 μl of phosphate-buffered saline (PBS, G4202, Servicebio, Wuhan, China) or LA (≥98%, 62230, Sigma-Aldrich, Wyoming, United States), respectively. The IOF procedure was conducted as previously reported (Chen et al., 2020). In brief, the site for injection was found by illumination (about one-third from the sharp end of the egg) and disinfected with 75% ethanol before injection. A small hole (0.8 mm in diameter) was drilled on the eggshell by a hand-hold pearl driller. The sterile disposable 25.0 × 0.6 mm needle was attached to a 1.0 ml syringe, which was replaced after each egg injection. The hole was sealed with medical adhesive tape (1.0 × 1.0 cm^2^) immediately after injection, and the egg was transferred to the incubator. All eggs were kept outside the incubator for less than 30 min during the injection process. Twelve eggs were selected to collect samples (two embryos per replicate, *n* = 6) on E28 and E31.

### Sample Collection

Samples of serum and liver were pooled together for each replicate in Experiment 1 (five embryos) and Experiment 2 (two embryos). Blood samples were collected from the umbilical vein of the embryos using glass Pasteur pipettes (7 × 150 mm). The tip of a glass Pasteur pipette was melted on the outer flame of an alcohol lamp and then withdrawn with a forcep to make a needle <0.3 mm in diameter. Blood samples were collected from the jugular vein of ducklings using disposable syringes at hatch. The serum was separated by centrifugation (1–14, Sigma, Germany) at 664 × *g* for 10 min at room temperature and then stored at −20°C for biochemical index analysis. The liver samples were cleaned of extraneous tissues, rinsed with ice-cold PBS, and then frozen in liquid nitrogen and stored at −80°C for analysis of biochemical index and relative expression of gene mRNA and protein.

### Biochemical Analysis

The serum glucose was measured using an automatic biochemical analyzer (Roche Cobas C702, Basel, Switzerland). The contents of glycogen, pyruvate, lactate, triacylglycerol (TG), and cholesterol (CHO) in serum or liver were measured according to the manufacturer’s instructions for each assay kit (Nanjing Jiancheng Bioengineering Institute, Nanjing, China). The serum contents of adenosine phosphate and coenzyme were measured using the ELISA assay kits as follows (Shanghai Enzyme-linked Biotechnology Co., Ltd., Shanghai, China): ATP, adenosine diphosphate (ADP), adenosine monophosphate (AMP), acetyl coenzyme A (acetyl CoA), and malonyl coenzyme A (malonyl CoA). Hepatic nicotinamide adenine dinucleotide (NAD) contents were measured using the assay kit (Beyotime Biotechnology, Shanghai, China) including NADH (reduced form) and NAD^+^ (oxidized form).

### Fatty Acids Analysis

FA profiles were determined by using gas chromatography (7890A, Agilent Technologies, Santa Clara, CA, United States). Lipids from liver tissues (Experiment 2) were extracted with chloroform and methanol (2:1, vol/vol), and the separated lipid fraction was converted to FA methyl esters (FAMEs) by saponification using 0.5 M KOH-methanol, followed by methylation with 200 μl boron trifluoride diethyl etherate (B104430, Sigma-Aldrich). Next, the FAMEs were mixed with N-hexane and saturated sodium chloride solution, and the mixture was shaken for 10 min and centrifuged at 1,200 × *g* for 10 min. The upper phase was collected and dried with sodium sulfate for gas chromatographic analysis. The FAMEs were identified by comparing retention times to FAME standards (CRM47885, Sigma-Aldrich). The results of FA composition were reported as the percentage of total FA.

### Gene mRNA Relative Expression Analysis

The total RNA isolation and the real-time quantitative procedure were conducted as previously reported (Kang et al., 2017; Madkour et al., 2021). Total RNA was extracted from the liver samples according to reagent protocols using a Trizol reagent (Invitrogen, Carlsbad, USA) followed by purification of total RNA using the kit with DNase treatment (Magen, Guangzhou, China). The RNA quality and quantity were determined using agarose gel electrophoresis (4.5%) and NanoDrop 2000 (Thermo Scientific, Wilmington, DE, USA). One μg of total RNA was converted into cDNA by reverse transcription as described in Primer Script RT Reagent Kits (TaKaRa, Dalian, China). Primer sequences were obtained from GenBank (Table 1) and designed and synthesized by Sangon Biotechnology Co., LTD (Shanghai, China). The PCR products were analyzed by using agarose gel electrophoresis (4.5%). Each qPCR reaction had a final volume of 10 μl of the reaction mixture, which consisted of 5 μl SYBR Green Realtime PCR Master Mix kit (QPK-201, TOYOBO, Osaka, Japan) with 3.2 μl DNase/RNase-Free water, 0.4 μl forward and reverse specific primers for each gene and 1 μl of cDNA template. Real-time quantitative PCR was performed using the detection system (Applied Biosystems QuantiStudio 7 Flex, Life Technologies, Carlsbad, CA, United States). The following cycling conditions were used: 95°C for 1 min, followed by 40 cycles each at 95°C for 15 s, 60°C for 15 s, and 72°C for 45 s. Melt-curve analysis was performed to verify the specificity of qPCR-amplified products. The quantification of the mRNA expression was calculated by the comparative CT method (2^−ΔΔCT^; Livak and Schmittgen, 2001).

**Table 1:** Forward and reverse primer sequences for PCR analysis.

Target genes	Forward primer (5'→3')	Reverse primer (5'→3')	Genebank accession no.	Product size (bp)
*PRKAA1*	GCGGCGGCGGATAAACAGAAG	CATGCTTGCCAACCTTGACTTTGC	XM_027447031.2	112
*PC*	CAACTACCTGCCCAACCTGCTG	GGCTGTATTTGGTGCGTGTAGGG	NM_205471.1	119
*PCK*	GAGCCATTGCCACCAGGAGTAAC	GCAGAACCGTGAGTTGGGATGAG	XM_021276483.2	99
*PFKFB2*	ACTTCTTCAGGCACGATAACAAGGAG	CGAGTTGTGTTGGTCGCATCAAAC	XM_038168182.1	136
*G6PC1*	CTGGCTCAACCTCGTCTTCAAGTG	GGCGTTGCTGTAGTAGTTGGTCTC	XM_027445511.2	85
*HKDC1*	AGGCAAGCCAGCATTGACAAGG	TCCCTCAGCATATCAACAACATCTTCC	XM_013092011.4	98
*PFKL*	GTGGGTGCCGTGAGAGAAGTTG	TGTAGAGGAACTCGGTGGTGTAGTG	XM_038183738.1	84
*PKM*	AGCCAACCATTGCGAGGAACAC	GTGGGTGCCGTGAGAGAAGTTG	XM_038184779.1	131
*FASN*	TCTCTGCCATCTCCCGAACTTCC	TTAGCCACTGTGCCAACTCAAGC	XM_027471234.2	96
*SCD1*	AGTTCTCCTCCGCTTCCAGC	TTCTCCATGACGGCATCCCC	XM_027460089.2	82
*PPARα*	ACCATCCTGATGATACCTTCCTCTTCC	AAGTTGAGCATGTTCTGTGACAAGTTG	NM_001310383.1	86
*RXRα*	TGCGAGCCATTGTCCTCTTCAAC	GATGCGTACACCTTCTCCCGTAAC	XM_027471073.2	88
*β-actin*	TACGCCAACACGGTGCTG	GATTCATCATACTCCTGCTTG	NM_00131042.1	215

*PRKAA1, protein kinase AMP-activated catalytic subunit alpha 1; PC, pyruvate carboxylase; PCK, phosphoenolpyruvate carboxykinase; PFKFB2, 6-phosphofructo-2-kinase; G6PC1, glucose-6-phosphatase; HKDC, hexokinase; PFKL, phosphofructokinase; PKM, pyruvate kinase M1/2; FASN, fatty acid synthase; SCD1, stearoyl-CoA desaturase; PPARα, peroxisome proliferator-activated receptor alpha; RXRα, retinoid X receptor alpha.*

### Western Blotting Analysis

The liver samples collected on E16, E22, E28, and hatch were used for western blotting analysis. The tissues were lysed with ice-cold RIPA lysis buffer containing 1 mmol/l phenylmethylsulfonyl fluoride. The lysates were placed on ice for 10 min and the homogenate was centrifuged at 12,000 × *g* for 5 min at 4°C. The protein concentration in the collected supernatant was measured using the bicinchoninic acid (BCA) assay kit (Beyotime Biotechnology, Shanghai, China). The protein supernatant was mixed with loading buffer, denatured for 5 min by heat shock at 95°C, and then stored at 4°C for western blotting analysis. Proteins were separated *via* an electrophoretic SDS-PAGE on 10% gel. Next, proteins were transferred to a nitrocellulose membrane with Trans-Blot, and the membrane was blocked with a buffer containing 5% skim milk. Membranes were incubated overnight at 4°C with rabbit monoclonal antibodies for adenosine monophosphate-activated protein kinase alpha 1 (AMPKα1, ab32047, Abcam, Cambridge, England), glucose-6-phosphatase (G6PC1, ab243319, Abcam), and β-actin (66009-1-Ig, Proteintech, Chicago, United States). Then, membranes were incubated with HRP goat anti-mouse IgG (SA00001-1, Proteintech) or HRP goat anti-rabbit IgG (SA00001-2, Proteintech) at room temperature. Proteins of interest were diluted in buffer with enhanced chemiluminescence solution. The bands were detected using a chemiluminescence imaging system (ChemiScope6100, Qinxiang, Shanghai China). The density of bands was determined using ImageJ software and the results were expressed as the intensity signal in arbitrary units after normalization.

### Statistical Analysis

Data from Experiment 1 were statistically analyzed using the PROC GLM procedures for one-way ANOVA by Statistical Analysis System v9.2 (SAS Inst. Inc., Cary, NC, United States). When significant differences were found (*p* < 0.05), Duncan’s multiple comparisons test was performed and orthogonal polynomial contrasts were used to identify the form of the effect (linear or quadratic) over time (incubation days). Data from Experiment 2 on each sampling time were analyzed with a *t*-test using the PROC Ttest procedure in SAS v9.2, and differences were considered to be significant at *p <* 0.05.

## Results

### Biochemical Index Related to Glucose Metabolism (Experiment 1)

**Figure 1:**
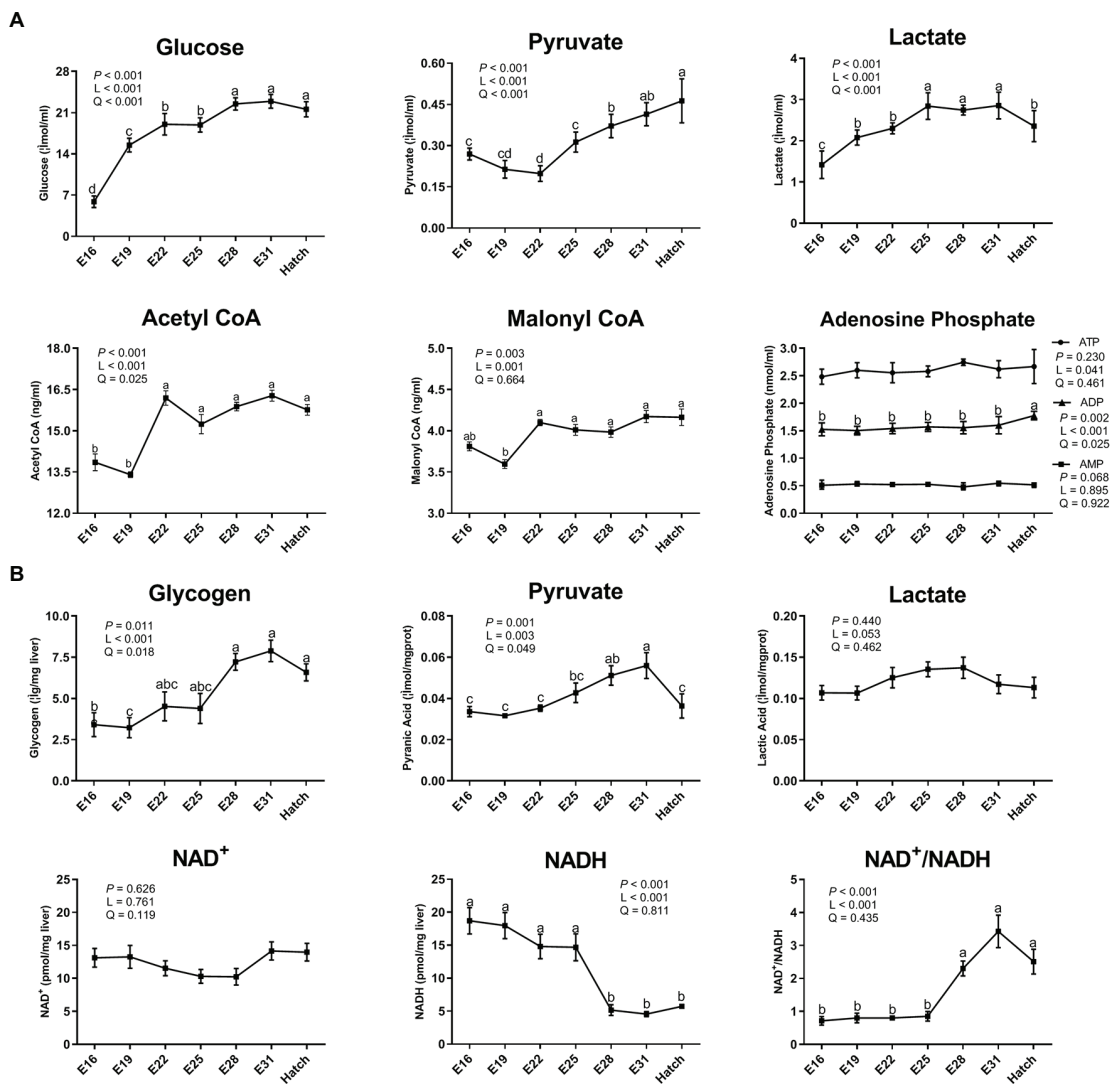
Dynamic changes of serum and hepatic biochemical index in the avian model. **(A)** serum biochemical index; **(B)** hepatic biochemical index. Abbreviations: CoA, coenzyme A; ATP, adenosine triphosphate; ADP, adenosine diphosphate; AMP, adenosine monophosphate; NAD, nicotinamide adenine dinucleotide; NAD^+^, the oxidated form of NAD; NADH, the reduced form of NAD. Data are expressed as mean ± SEM (*n* = 6), and values on the same line with different lowercase letters indicated statistically significant differences (one-way ANOVA, *P* < 0.05). L, linear; Q, quadratic.

The serum contents of glucose, pyruvate, and lactate increased (*p* < 0.05) in a linear and quadratic manner during embryogenesis (Figure 1A), reaching plateau periods during E28 - hatch, E31 - hatch, and E25–E31, respectively. The serum contents of ADP, acetyl CoA, and malonyl CoA increased (*p* < 0.05) linearly or quadratically (Figure 1A). Hepatic glycogen and pyruvate increased (*p* < 0.05) in a linear and quadratic manner, with the peak period of E28 - hatch or E28–E31, respectively (Figure 1B). As incubation day increased, hepatic NADH content decreased (*p* < 0.05) linearly and the ratio of NAD^+^ to NADH increased (*p* < 0.05) linearly, with maximal changes observed at the period of E28 - hatch (Figure 1B).

### Hepatic Gene and Protein Expression Related to Glucose Metabolism (Experiment 1)

**Figure 2:**
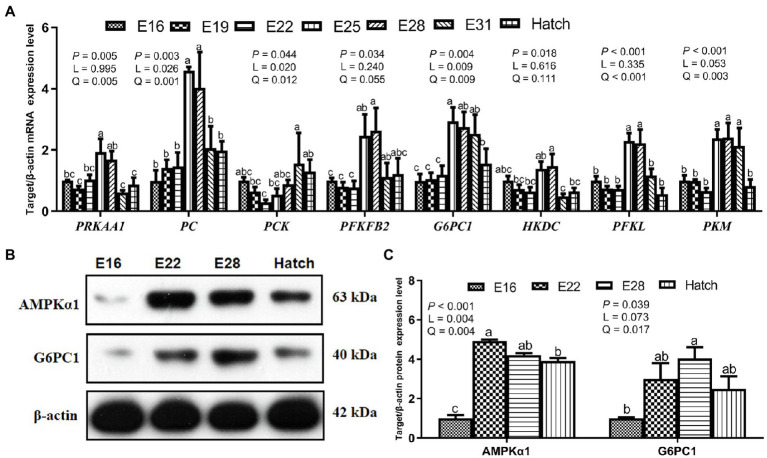
Hepatic gene and protein expression related to glucose metabolism in the avian model. **(A)** Hepatic gene mRNA relative expression related to glucose metabolism in the avian model; **(B,C)** The expression of proteins related to glucose metabolism in the liver of avian embryos. *PRKAA1*, protein kinase AMP-activated catalytic subunit alpha 1; *PC*, pyruvate carboxylase; *PCK*, phosphoenolpyruvate carboxykinase; *PFKFB2*, 6-phosphofructo-2-kinase; *G6PC1*, glucose-6-phosphatase; *HKDC*, hexokinase; *PFKL*, phosphofructokinase; *PKM*, pyruvate kinase M1/2; AMPKα1, AMP-activated protein kinase alpha 1. Data are expressed as mean ± SEM, and bars with different lowercase letters indicated statistically significant differences (one-way ANOVA, *p <* 0.05). L, linear; Q, quadratic.

Hepatic mRNA expression related to energy regulation, gluconeogenesis, and glycolysis of protein kinase AMP-activated catalytic subunit alpha 1 (*PRKAA1*), pyruvate carboxylase (*PC*), phosphoenolpyruvate carboxykinase (*PCK*), 6-phosphofructo-2-kinase (*PFKFB2*), *G6PC1*, hexokinase (*HKDC*), phosphofructokinase (*PFKL*), and pyruvate kinase M1/2 (*PKM*) increased (*p* < 0.05) linearly or quadratically in response to the increased incubation day, and the plateau period was shown between E25 and E31 (Figure 2A). Hepatic protein expression of AMPKα1 and G6PC1 increased (*p* < 0.05) linearly or quadratically, and the higher level was presented between E22 and E28 (Figures 2B,C).

### Effect of IOF LA on Biochemical Index and Fatty Acids (Experiment 2)

**Figure 3:**
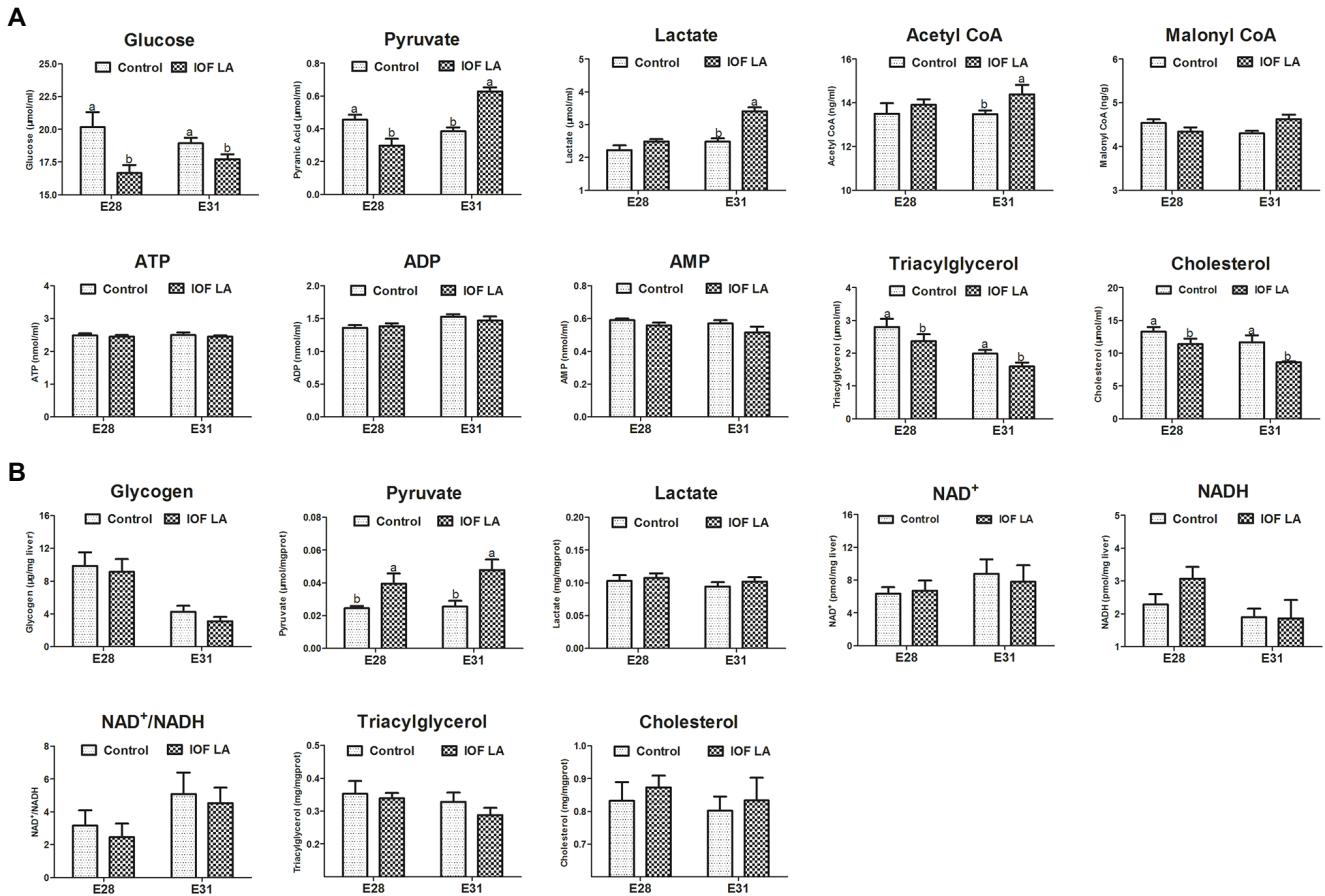
Effect of *in ovo* feeding linoleic acid on the changes of biochemical index in the avian model. **(A)** serum biochemical index; **(B)** hepatic biochemical index. Control group was *in ovo* injected with PBS, and IOF LA was the treatment group *in ovo* feeding linoleic acid on E25. Abbreviations: CoA, coenzyme A; ATP, adenosine triphosphate; ADP, adenosine diphosphate; AMP, adenosine monophosphate; NAD, nicotinamide adenine dinucleotide; NAD^+^, the oxidated form of NAD; NADH, the reduced form of NAD. Data are expressed as mean ± SEM (*n* = 6), and bars on the same sampled day with different lowercase letters indicated statistically significant differences (*t*-test, *p* < 0.05).

Compared to the control group, IOF LA group decreased (*p* < 0.05) serum contents of glucose, TG, and CHO on E28 and E31, and serum pyruvate content on E28 (Figure 3). IOF LA group increased (*p* < 0.05) serum contents of pyruvate, lactate, and acetyl CoA on E31, and hepatic pyruvate content on E28 and E31 (Figures 3A,B). For FA profiles, IOF LA group increased (*p* < 0.05) hepatic stearic acid (C18:0) and saturated FAs (SFAs) on E28, and myristic acid (C14:0) and SFAs on E31, while IOF LA group decreased (*p* < 0.05) hepatic oleic acid (C18:1n-9), unsaturated FAs (UFAs), and the ratio of UFAs to SFAs on E28 by comparison to the control group (Table 2).

**Table 2:** Effect of *in ovo* feeding linoleic acid on the changes of hepatic fatty acid composition in the avian model (%).

Item	E28			E31		
	Control	IOF LA	SEM	Control	IOF LA	SEM
Myristic acid	C14:0	5.77	5.41	0.341	5.08^b^	6.96^a^	0.379
Myristoleic acid	C14:1	0.57	0.60	0.067	0.67	0.77	0.047
Palmitic acid	C16:0	16.96	15.94	0.478	16.91	15.60	0.522
Palmitoleic acid	C16:1	0.59	0.58	0.052	0.47	0.33	0.044
Stearic acid	C18:0	12.65^b^	16.42^a^	0.715	16.28	17.15	0.476
Oleic acid	C18:1n-9	30.18^a^	27.33^b^	0.831	26.86	25.84	0.883
Linoleic acid	C18:2n-6	8.68	8.96	0.294	8.09	8.23	0.289
Eicosatrienoic acid	C20:3n-6	0.34	0.41	0.021	0.41	0.40	0.039
Eicosatrienoic acid	C20:3n-3	14.14	15.40	0.646	15.40	16.31	0.779
Nervonic acid	C24:1n-9	0.89	0.67	0.105	0.95	0.86	0.041
Docosahexaenoic acid	C22:6n-3	6.21	6.29	0.524	6.49	5.47	0.360
MUFAs		31.73	29.19	0.818	28.87	27.80	0.922
PUFAs		30.01	31.06	0.616	30.39	30.42	0.976
n-6 PUFAs		9.03	9.37	0.294	8.50	8.64	0.290
n-3 PUFAs		20.98	21.70	0.692	21.89	21.78	1.029
n-6/n-3		0.43	0.44	0.025	0.41	0.40	0.032
UFAs		61.74^a^	60.25^b^	0.441	59.25	58.21	0.399
SFAs		36.16^b^	37.77^a^	0.434	38.26^b^	39.70^a^	0.432
UFAs/SFAs		1.71^a^	1.60^b^	0.030	1.55	1.47	0.026

Control group was *in ovo* injected with PBS, and IOF LA was the treatment group *in ovo* feeding linoleic acid on E25. MUFAs are the sum of monounsaturated fatty acids that include C14:1, C16:1, C18:1n-9, and C24:1n-9. PUFAs are the sum of polyunsaturated fatty acids that include C18:2n-6, C20:3n-6, C20:3n-3, and C22:6n-3. N-6 PUFAs are the sum of n-6 polyunsaturated fatty acids that include C18:2n-6 and C20:3n-6. N-3 PUFAs are the sum of n-3 polyunsaturated fatty acids that include C20:3n-3 and C22:6n-3. UFAs are the sum of unsaturated fatty acids that include MUFAs and PUFAs. SFAs are the sum of saturated fatty acids that include C14:0, C16:0, and C18:0. Data are expressed as mean ± SEM (*n* = 6), and data at the same sampled day on the same line with different lowercase letters indicated statistically significant differences (*t*-test, *p* < 0.05).

### Effect of IOF LA on Hepatic Gene Relative Expression (Experiment 2)

**Figure 4:**
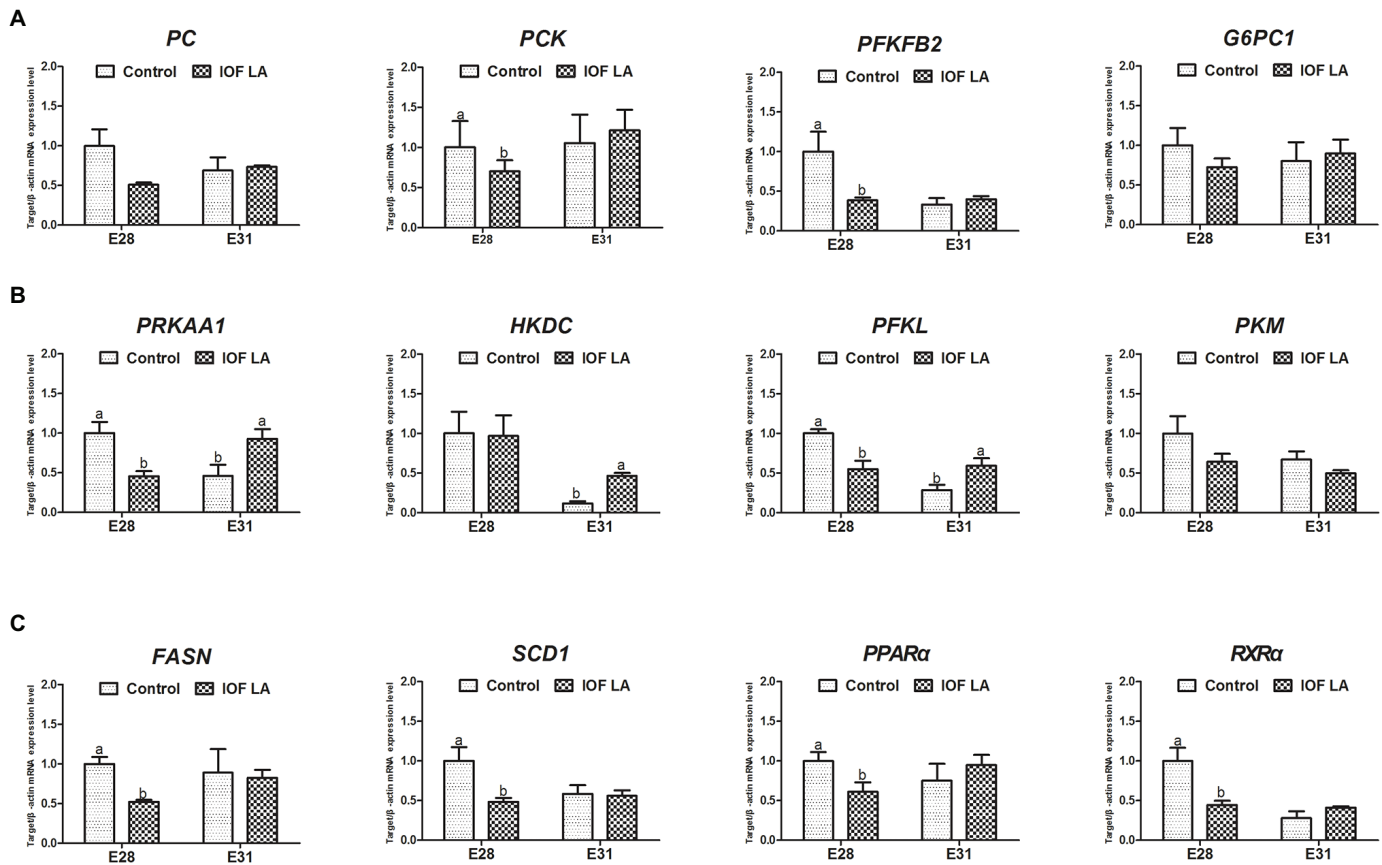
Effect of *in ovo* feeding linoleic acid on the gene relative expression in the avian model. Control group was *in ovo* injected with PBS, and IOF LA was the treatment group *in ovo* feeding linoleic acid on E25. **(A)** gene expression related to gluconeogenesis: *PC*, pyruvate carboxylase; *PCK*, phosphoenolpyruvate carboxykinase; *PFKFB2*, 6-phosphofructo-2-kinase; *G6PC1*, glucose-6-phosphatase; **(B)** gene expression related to energy homeostasis and glycolysis: *PRKAA1*, protein kinase AMP-activated catalytic subunit alpha 1; *HKDC*, hexokinase; *PFKL*, phosphofructokinase; *PKM*, pyruvate kinase M1/2; **(C)** gene expression related to fatty acid synthesis and oxidation: *FASN*, fatty acid synthase; *SCD1*, stearoyl-CoA desaturase; *PPARα*, peroxisome proliferator-activated receptor alpha; *RXRα*, retinoid X receptor alpha. Data are expressed as mean ± SEM (*n* = 6), and bars on the same sampled day with different lowercase letters indicated statistically significant differences (*t*-test, *p* < 0.05).

IOF LA group decreased (*p* < 0.05) hepatic mRNA expression of *PRKAA1, PC, PCK, PFKFB2, PFKL, FASN, SCD1, PPARα,* and *RXRα* on E28, and increased (*p* < 0.05) mRNA expression of *PRKAA1, HKDC,* and *PFKL* on E31 (Figure 4).

## Discussion

Hepatic glucose metabolism is essential for energy homeostasis by balancing glucose storage and utilization during incubation. The blood glucose supply and muscle and hepatic glycogen storage were mainly mediated by the metabolic pathway of gluconeogenesis in embryogenesis (De Oliveira et al., 2008; Guo et al., 2013). In addition, both blood glucose and hepatic glycogen levels are considered as the criterion in assessing embryonic energetic status based on the data examined in carbohydrates metabolism (Christensen et al., 1999, 2000). Previous studies demonstrated that blood glucose concentration is positively associated with embryonic survival and hatchling weight (Christensen et al., 2000). Therefore, the avian embryo in the final stage of incubation has been considered as a suitable model for assessing the connection between glucose metabolism and energy status. In the present study, serum glucose and hepatic glycogen contents increased as incubation proceeded from E16 to hatch, and the plateau period was shown on the last week of the incubation (E28 - hatch). These parallel changes of serum glucose and hepatic glycogen were also confirmed in chick embryos (Latour et al., 1996; Christensen et al., 2001; Roy et al., 2013a). The rate-limiting enzymes of PC, PCK, PFKFB2, and G6PC1 are responsible for the irreversible catalytic reactions in gluconeogenesis (Shen and Mistry, 1979; van Schaftingen and Gerin, 2002; Chesney, 2006). Hepatic gene mRNA expression of *PRKAA1, PC, PCK, PFKFB2,* and *G6PC1,* and the relative protein expression of G6PC1 increased from E16 to hatch, and the maximal values were observed between E22 and E31. These results could partly explain the increasing serum glucose concentration and hepatic glycogen content during embryonic development. The avian embryo begins anaerobic catabolism of glucose before hatch, and glucose is metabolized into the highly versatile metabolite pyruvate (De Oliveira et al., 2008). And then, pyruvate can be anaerobically oxidized to lactate, accompanied by the conversion of NADH to NAD^+^ (Bar-Even et al., 2012; Luengo et al., 2021). In the current study, glycolysis was enhanced as indicated by an increase in pyruvate and lactate contents in serum or liver and a decrease in hepatic NADH content during the last period of incubation (E28 - hatch). Glycolysis supplies the ATP molecules produced by a reversible reaction (2ADP ↔ ATP + AMP; Noor et al., 2010; Schormann et al., 2019). The increase in ADP content implied that ATP was hydrolyzed to generate energy for the maintenance of embryonic growth and development. It’s suggested that glycolysis provides an additional energy source (except for FA oxidation) for the greater demands during the later period of incubation, especially during internal piping (break the air cell by the beak) and external piping (break the eggshell) periods (Moran, 2007; De Oliveira et al., 2008). Glycolysis is regulated by the key enzymes of HKDC, PFKL, and PKM (Bolaños et al., 2010). Hepatic gene mRNA expression of *HKDC, PFKL,* and *PKM* increased during embryonic development, and the plateau period was achieved between E25 and E31. A similar pattern was observed in chick and pigeon embryos reported previously (Roy et al., 2013b; Peng et al., 2018; Wan et al., 2018). Then, the increased protein expression level of AMPKα1 was observed in liver during E22 - E28. It’s implied that the AMPK pathway is activated by falling energy status and promotes ATP production *via* increasing catabolism as well as switching off biosynthetic pathways (Hardie et al., 2012; Hardie, 2015). Briefly, these results suggested that the glucose homeostasis turnover between gluconeogenesis and glycolysis enhanced energy generation to meet the high energy requirements during embryonic development and as fuel storage for survival at the early post-hatch period (Li et al., 2008; Payne et al., 2019).

Previous studies have demonstrated that LA reduces glucose concentration in the diverse cell, animal, and human models (Conde-Aguilera et al., 2012; Matravadia et al., 2016; Andersson-Hall et al., 2018; Holmäng et al., 2018). In the present study, the intervention of exogenous LA led to a decrease in serum contents of TG and CHO, and then reduced the concentration of glycerol, which was derived from TG hydrolysis and the main precursor of gluconeogenesis in late-term avian embryos (Sunny and Bequette, 2011; Neves et al., 2017; Xue et al., 2017). Moreover, IOF LA inhibited gluconeogenesis on E28 as displayed by a decrease in serum glucose concentration and gene expression of *PC*, *PCK*, and *PFKFB2.* However, IOF LA increased stearic acid (C18:0) and decreased oleic acid (C18:1n-9) in liver, due to the weakened activity of desaturases responsible for the synthesis of UFAs, especially the Δ9-desaturase activity (Kouba and Mourot, 1998; Bee, 2000; Novak et al., 2012; Lounis and Bergeron, 2017; Piccinin et al., 2019). Hepatic mRNA expression of *FASN, SCD1, PPARα,* and *RXRα* related to FA synthesis and oxidation was downregulated on E28. It’s implied that FA oxidation could not satisfy all the energy demands for the embryos as the internal oxygen concentration became limited. In addition, IOF LA enhanced glycolysis on E31 as indicated by an increase in serum pyruvate and lactate contents, as well as the upregulation of hepatic gene expression of *HKDC* and *PFKL*. The synthesis of serum glucose and hepatic glycogen was suppressed, combined with the enhanced glycolysis, which led to a reduced energy fuel storage for late embryonic development. Hence, the embryonic mortality in IOF LA group (24.27%) was increased by 8% compared to the control group (16.67%). Similarly, *in vivo* studies showed that elevated maternal dietary LA (or conjugated LA) reduced fetal survival and increased embryonic mortality in rat or chicken models (Leone et al., 2010; Shrestha et al., 2019). It was concluded exogenous LA could inhibit hepatic gluconeogenesis and enhanced glycolysis, thus leading to the impairment of glucose homeostasis and energy status during embryonic development.

## Conclusion

In summary, hepatic gluconeogenesis and glycolysis were enhanced to meet increasing energy demands during the late embryonic period, as evidenced by increasing contents of glucose, glycogen, pyruvate, and lactate in serum or liver, as well as hepatic target gene and protein expressions. IOF LA could inhibit gluconeogenesis and enhance glycolysis, leading to impaired glucose homeostasis and energy status in the developing embryos.

## Data Availability Statement

The original contributions presented in the study are included in the article/Supplementary Material, further inquiries can be directed to the corresponding authors.

## Ethics Statement

The animal study was reviewed and approved by Animal Care and Use Committee of South China Agricultural University.

## Author Contributions

XZ designed this study, carried out the experiments and measurements, and drafted the manuscript. QW, WZ, CL, and LH helped to analyze the experiment traits. WZ, XZ, and XH assisted with the incubation trial. HY and WW helped with the data analysis. LY and YZ participated in the study’s design, coordination, and manuscript writing. All authors read and approved the final version of the manuscript.

## Funding

This study was sponsored by Provincial Natural Science Foundation for Cooperation with WENS Group (2019B1515210031), National Natural Science Foundation of China (3197200131 and 31802080), Provincial Rural Revitalization Foundation of China (F21125), and China Agriculture Research System of MOF and MARA (CARS-42-15).

## Conflict of Interest

CL, XZ, WX, and XH, are employed by Wen’s Food Group Co. Ltd.

The remaining authors declare that the research was conducted in the absence of any commercial or financial relationships that could be construed as a potential conflict of interest.

## Publisher’s Note

All claims expressed in this article are solely those of the authors and do not necessarily represent those of their affiliated organizations, or those of the publisher, the editors and the reviewers. Any product that may be evaluated in this article, or claim that may be made by its manufacturer, is not guaranteed or endorsed by the publisher.

## Abbreviations

ADP: Adenosine diphosphate
AMP: Adenosine monophosphate
AMPK: Adenosine monophosphate-activated protein kinase
ATP: Adenosine triphosphate
CHO: Cholesterol
CoA: Coenzyme A
FA: Fatty acid
FAMEs: Fatty acid methyl esters
HKDC: Hexokinase
IOF: *In ovo* feeding
LA: Linoleic acid
NAD: Nicotinamide adenine dinucleotide
PBS: Phosphate-buffered saline
PC: Pyruvate carboxylase
PCK: Phosphoenolpyruvate carboxykinase
PFKFB2: 6-Phosphofructo-2-kinase
PFKL: Phosphofructokinase
PKM: Pyruvate kinase M1/2
PRKAA1: Protein kinase AMP-activated catalytic subunit alpha 1
RT-qPCR: Real-time quantitative PCR
SFAs: Saturated fatty acids
TG: Triacylglycerol
UFAs: Unsaturated fatty acids
